# Modeling the Prospective Relationships of Impairment, Injury Severity, and Participation to Quality of Life Following Traumatic Brain Injury

**DOI:** 10.1155/2013/102570

**Published:** 2013-10-02

**Authors:** Ryan J. Kalpinski, Meredith L. C. Williamson, Timothy R. Elliott, Jack W. Berry, Andrea T. Underhill, Philip R. Fine

**Affiliations:** ^1^Department of Educational Psychology, 4225 TAMU, Texas A&M University, College Station, TX 77845-4225, USA; ^2^Samford University, Birmingham, AL 35229, USA; ^3^Injury Control Research Center, University of Alabama at Birmingham, Birmingham, AL 35294, USA

## Abstract

Identifying reliable predictors of positive adjustment following traumatic brain injury (TBI) remains an important area of inquiry. Unfortunately, much of available research examines direct relationships between predictor variables and outcomes without attending to the contextual relationships that can exist between predictor variables. Relying on theoretical models of well-being, we examined a theoretical model of adjustment in which the capacity to engage in intentional activities would be prospectively associated with greater participation, which in turn would predict subsequent life satisfaction and perceived health assessed at a later time. Structural equation modeling of data collected from 312 individuals (226 men, 86 women) with TBI revealed that two elements of participation—mobility and occupational activities—mediated the prospective influence of functional independence and injury severity to optimal adjustment 60 months following medical discharge for TBI. The model accounted for 21% of the variance in life satisfaction and 23% of the variance in self-rated health. Results indicate that the effects of functional independence and injury severity to optimal adjustment over time may be best understood in the context of participation in meaningful, productive activities. Implications for theoretical models of well-being and for clinical interventions that promote adjustmentafter TBI are discussed.

## 1. Introduction

 Reliable predictors of quality of life following traumatic brain injury have proven difficult to identify, prompting some observers to raise concerns about the clinical value of this research [1]. Injury severity, in particular, is inconsistently predictive of subsequent life satisfaction following TBI [1–3]. A recent panel concerned with outcomes after TBI asserted that indicators of participation—such as social integration, mobility, and community and vocational activity—consistently evince stronger associations with quality of life following TBI [4]. In many respects, participation constitutes an important rehabilitation outcome because it embodies the degree to which a person resumes an active role in valued personal and social pursuits [4]. Its relationship to personal adjustment supports the premium International Classification of Functioning, Disability, and Health places on participation [5]. 

 Recent conceptualizations of optimal adjustment following traumatically acquired disability emphasize the array of and dynamic relationships between personal and environmental characteristics that shape well-being over time [6]. Although functional impairments and injury severity may not directly influence life satisfaction, these factors impact other variables that do. For example, functional impairments can limit activities following TBI, and we know that participating in personally fulfilling activities is a vital component of well-being among people in general, [7] and restrictions in activity are association with distress and depression among those with chronic health conditions [8]. Active participation in desired pursuits increases the likelihood of positive emotional experiences that facilitates personal resilience, social connections, and life satisfaction among people in general [9, 10] and among individuals with acquired disabilities [11, 12]. The engagement in *intentional* activities—ones that reflect behavioral, volitional, and cognitive and goal-directed effort—is a significant determinant of happiness and life satisfaction, and its influence is greater than characteristics that reflect a demographic status or circumstance (e.g., socioeconomic status) [13]. 

 Individuals with greater functional impairment following TBI often experience greater difficulties in participating in meaningful activities after discharge. Over time, the resulting lack of participation then compromises quality of life [14]. In contrast, those who have less functional impairment are more likely to be involved in intentional pursuits, participate in desired activities, and consequently experience a higher quality of life over time. The relationships between functional abilities and injury severity are best examined in contextual analyses that take into account hypothesized associations between predictor and mediating variables in the prospective prediction of quality of life. 

More sophisticated analyses, informed by structural equation models, demonstrate that personal characteristics [15] have direct effects on participation after discharge, and there is evidence that participation can mediate the predictive association between functional impairments and quality of life over time. For example, in a study of 144 persons with traumatic spinal cord injuries, Erosa and colleagues [16] found that the prospective relationships of functional impairment to life satisfaction and self-perceived health were mediated by participation. Greater functional ability predicted greater participation three years later as hypothesized, which in turn predicted higher life satisfaction and better health status a year later. However, functional impairment had no direct influence on either outcome variable. Thus, the prospective relationship of functional impairment to life satisfaction and self-rated health was best understood in the context of participation. 

 We conducted the present study to examine the influence of functional impairment and injury severity on participation and how these factors prospectively predict elements of quality of life. We studied individuals who were part of a larger, longitudinal study of adjustment five years following medical discharge for a traumatic, disabling injury. Reasoning from relevant research and from our current understanding of participation on quality of life following disability, we assumed that functional impairments and injury severity would negatively affect participation in desired activities over time. Participation would then, in turn, significantly predict life satisfaction and self-reported health status—two important aspects of quality of life—assessed a year later. We utilized a contextual analytic model that would reveal the degree to which participation would mediate the possible influence of functional impairment and injury severity on life satisfaction and self-perceived health status over time. 

## 2. Materials and Methods

Participants were part of a larger longitudinal study conducted by the Injury Control Research Center (ICRC) at the University of Alabama at Birmingham. Prospective participants included persons who had traumatically incurred TBI and who had been discharged from a subset of nine hospitals surrounding north-central Alabama. Participants were identified from medical records at acute care hospitals and were contacted at twelve-month post-discharge to participate in the study. Individuals were invited to participate if they (1) were residents of and injured in Alabama; (2) were at least eighteen years old when injured; (3) were inpatients at an acute care hospital for three or more days; (4) were discharged alive from an acute care hospital between October 1, 1989 to September 30, 1992; and (5) consented to participate in regular follow-up interviews by telephone conducted by ICRC personnel. TBI was determined by the following ICD9 diagnosis codes in the medical record at the time of discharge from acute care: 800.00–800.9, 801.0–801.9, 803.0–803.9, 851.0–851.9, 852.0–852.5, 853.0, 853.1, 854.0, and 854.1. 

Eligible persons were contacted by mail at 12-month postdischarge to participate in the study. Preaddressed postcards containing consent forms were included. If the consent form was not returned by mail, ICRC personnel contacted eligible persons by phone to explain the study in greater detail. Persons contacted by ICRC personnel provided informed consent over the telephone. Data was collected from all consenting persons by a trained interviewer. Interviews were conducted with participants' spouses, caregivers, and close relatives when participants were unavailable or unable to answer questions over the telephone. Additional demographic and clinical information was collected from acute care hospital records. 

Measures were administered to participants through telephone interviews and mailed self-report questionnaires at 12, 24, 48, and 60 months after discharge from acute care hospitals. Data were collected on demographic and social characteristics, rehabilitation services and outcomes, other medical services, overall health status, psychological and physical adjustment to TBI, and secondary complications following TBI. 

Of the 1,026 eligible persons with TBI contacted to participate, 609 individuals (435 men, 174 women) with TBI consented to participate. The present study included 312 participants (226 men, 86 women) with complete data for each variable at each time point for subsequent analysis. The mean age of participants in the sample was 36.8 years (SD = 15.9 years). Women were significantly older than the men at time of injury (men, M age = 34.1 years; women, M age = 43.6 years; *P* < 0.001). Most participants identified either as Caucasian (*n* = 226) or African American (*n* = 84).

### 2.1. Measures

#### 2.1.1. Injury Severity

The rating on Abbreviated Injury Scale (AIS) [17, 18] for the head region was used as an indicator of TBI severity [19]. The AIS provides an anatomical description of injury severity for six body regions, including the head, based on ordinal values ranging from *minor injury* (1) *to maximum injury or virtually unsurvivable* (6) [17, 19]. Trained raters utilized information from the ICD9 diagnosis codes in the acute medical record for AIS coding. AIS scores for each of the six body regions, including the head, were calculated through the use of ICDMAP, a computerized table that converts ICD-9-CM coded discharge diagnoses to AIS scores [18]. Over forty-two percent (42.7%) of the present sample had overall AIS head ratings indicative of moderate injury severity, 29.8% were rated as serious, 21.7% were rated as severe, and 5.8% were rated as critical. Comparisons of the AIS scores with the commonly used Glasgow Coma scores provided in the Brasure et al. report for the Agency for Healthcare Research and Quality [20] suggest that 42.7% of the sample experienced moderate TBIs (GCS scores of 9 to 12) and the majority experienced severe TBIs (GSC scores 3–8).

#### 2.1.2. Functional Independence

The Functional Independence Measure (FIM) [21] was used to assess functional abilities. The FIM is a self-report measure that contains 18 items with Likert-type rating scales ranging from need for *total assistance* (1) to *complete independence* (7) to complete activities of self-care, locomotion, sphincter control, social cognition, transfers, and communication. Higher scores on the FIM indicate greater functional independence. The FIM has acceptable reliability and validity for use in TBI rehabilitation [22]. The internal consistency of items on the FIM was 0.94 for the present sample. FIM items were linearized utilizing Rasch scaling procedures in order to increase item variability and ensure item quality, stability, and reliability [23, 24].

The FIM was administered by telephone in the 12th month of participation. Two components of the FIM were used in this study, the Cognitive FIM and the Motor FIM, to maximize our understanding of the influence of these functional abilities on subsequent participation and quality of life. 

#### 2.1.3. Participation

The Craig Handicap Assessment Reporting Technique (CHART) [25] was first administered to participants in the 48th month after discharge to assess participation. The CHART is a self-report measure of activity and participation in six broad domains. It was designed to assess participation restrictions among persons with severe disability, and it measures participation in a manner congruent with the WHO International Classification of Functioning model [20, 26]. As a self-report measure, the CHART assesses participation from the perspective of the individual, such that the individual's life context, circumstance and subjective experience are respected [27]. It is often recommended as a measure of participation among persons with TBI [4, 20]. We selected the Mobility, Social Integration, and Occupation Scales for use in the present study. Higher scores on these subscales indicate greater participation. 

 The Mobility (MOB) Scale contains nine items that require the respondent to indicate their ability to freely move around in their residence and in their community (e.g., “In a typical week, how many days do you get out of your house and go somewhere?”, “Can you enter and exit your home without any assistance from someone?”, “Can you use your transportation independently?”). The Occupation (OCC) Scale consists of seven items and measures time spent in recreational activities, home maintenance and household tasks, in paid work and in volunteer activities. The Social Integration (SI) Scale contains six items that ask the respondent to report the number of friends, relatives, and business associates with whom they interact at least once a month, how often they initiated conversations with strangers, and so forth. 

#### 2.1.4. Quality of Life

Two measures were used to assess distinct but related components of quality of life at 60-month post-discharge. The Life Satisfaction Index (LSI) [28] was used to assess life satisfaction. The LSI contains 20 statements regarding current life satisfaction to which they responded “agree” or “disagree.” The internal consistency of the items on the LSI was 0.85 for the present sample. The LSI has acceptable psychometric properties, and it is considered one of the best available measures of life satisfaction in health outcomes research [29]. Higher scores indicate greater life satisfaction. 

An indicator of health status was obtained with a single item, “In general, how would you rate your health at the present time?” [30]. Participants rated their response as 1 = *Excellent* (no health problems), 2 = *Good* (no major health problems, but a few minor health problems), 3 = *Fair* (several minor health problems), or 4 = *Poor* (major health problems affecting me daily). Lower scores indicate a more positive health status.

### 2.2. Data Analysis

Descriptive statistics were generated using SPSS version 20.0. Relationships among demographic characteristics and self-report variables were examined by calculating Pearson *r* correlation coefficients, *t*-tests, and Spearman's rho correlation coefficients. Univariate and multivariate normality are assumed in structural equation modeling (SEM) because nonnormal data can produce biased parameter estimates [31]. Univariate and multivariate normality were assessed through examination of the data for skewness, kurtosis, and outliers prior to SEM analyses. Additional data screening was conducted to eliminate participants with missing values on variables included in the SEM analyses. Therefore, all participants included in the SEM analyses had values for each variable within the specified model. 

 A structural equation model (SEM) was used to test the direct and indirect effects of head injury severity (AIS) during acute care, cognitive (COG) and motor (MOT) functional independence at 12-month post-discharge, and the participation variables of mobility, occupational activity, and social integration at 48-month post-discharge on life satisfaction (LSI) and self-rated health status (HEALTH) at 60-month post-discharge. SEM is a powerful technique for analyzing theory-driven contextual models that stipulate complex relationships between predictor and mediator variables in the prediction of important outcomes; the exploratory and confirmatory features of SEM facilitate an understanding of theorized relationships and provide empirical guidance for refinement [32]. 

**Figure 1:**
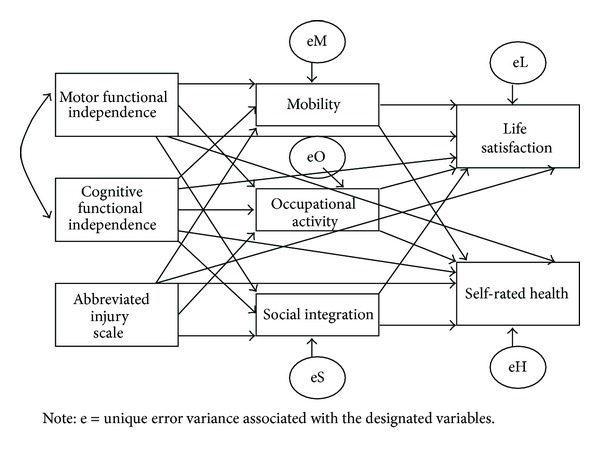
*A priori* path model of motor functional independence, cognitive functional independence, Abbreviated Injury Scale (head), mobility, occupational activity, social integration, life satisfaction, and self-rated health.

The theoretically derived path model in Figure 1 was analyzed by treating all variables as observed (e.g., measured) variables. Statistically significant paths were expected to proceed from the exogenous predictor variables (AIS, COG, and MOT) to the potentially mediating participation variables (mobility, occupation, and social integration) to the endogenous outcome variables (LSI, HEALTH). Both indirect and direct paths from exogenous variables to endogenous outcome variables were included in the theory-driven model.

 Mplus version 6.11 was used for all SEM analyses because of the program's ability to run bootstrapping techniques necessary for investigating mediation within the model. Path model analyses were conducted using a maximum likelihood estimator. Model fit was assessed using recommended model fit statistics including the *χ* ^2^ test, root-mean square error of approximation (RMSEA), and standardized root mean square residual (SRMR) [33, 34]. Adequate model fit is typically achieved when *χ* ^2^ tests of model fit are statistically nonsignificant, RMSEA scores are approximately less than 0.06, and SRMR scores are generally less than 0.08 [35]. These criteria were employed to evaluate adequate model fit in the present study.

 Bootstrap confidence intervals were computed using Mplus software to evaluate indirect effects (i.e., mediation) between variables [36]. Bootstrap analysis is a nonparametric resampling technique that does not invoke the assumption of normality of the sampling distribution [36–38]. Through a computationally intensive resampling procedure, bootstrapping provides empirical approximations of sampling distributions and confidence intervals. Indirect effects of mediating variables are estimated by the confidence intervals constructed through the bootstrapping procedure [36]. Without collecting new data, the bootstrap analysis may be considered the closest approximation to external replicability [39]. 

## 3. Results

Means, standard deviations, and correlations between the study variables are contained in Table 1. The Rasch-scaled Motor and Cognitive FIM scores were significantly associated with the participation and two outcome variables (life satisfaction and perceived health) in expected directions. The participation and outcome variables were also significantly correlated. Comparisons between the 312 participations with complete data included in Table 1 were significantly younger (M age = 36.8) and reported more functional independence (M total FIM raw score = 117.14) than those who were excluded due to missing data at any measurement occasion (M age = 39.9; M total FIM raw score = 110.71). The two groups did not differ by gender or ethnicity. 

Multivariate and univariate normality were assessed prior to SEM analyses. Identification of univariate outliers was accomplished through inspection of *z* score frequency distributions (e.g. |*z* | >3 represents univariate outliers) [31]. Upon inspection, five univariate outliers were detected in the data and converted to a value equal to the most extreme score within three standard deviations of the mean as suggested by Kline [31]. Mahalanobis distance criterion (*P* < 0.001) was used to identify multivariate outliers [31]. Three multivariate outliers were identified in the data, and the participants were subsequently removed from further analyses. Skewness and kurtosis were also assessed to determine normality, and all variables were within acceptable limits to proceed with the SEM analyses. SEM analyses were conducted with 309 participants.

 After reviewing the bivariate correlation matrix, the correlation between exogenous variables motor functional independence and cognitive functional independence was statistically significant (0.530, *P* < 0.01). Therefore, the covariance path between motor and cognitive functional independence was included in subsequent SEM analyses. 

### 3.1. Model Evaluation

 The theoretically derived path modal was evaluated for model fit. Fit statistics for the theoretically derived path model indicated poor fit for the data, *χ* ^2^ (3) = 113.832, *P* < 0.001 (RMSEA = 0.346, SRMR = 0.077). As a result, SEM analyses were conducted to examine the statistical significance of the paths within the theoretically derived path model. Various paths within the original model were removed and refitted to test an empirically derived model.

The corrected model excluded social integration as a predictor variable because there were no statistically significant paths between social integration to life satisfaction or self-rated health. Furthermore, the inclusion of social integration was excluded from the corrected model, because social integration hindered the model from reaching adequate fit. 

Other refinements to the model were required, including the removal of nonsignificant paths. All direct paths leading from the exogenous variables (AIS, COG, and MOT) to self-rated health status were not significant and were removed from the model. Two direct paths leading from exogenous variables (AIS, COG) to life satisfaction were statistically nonsignificant and were removed from the model. 

Additionally, the nonsignificant path from AIS to mobility and the path from mobility to life satisfaction were removed. Finally, a single path was inserted leading from mobility to occupational activity (unstandardized coefficient = 0.907, standardized coefficient = 0.489, *P* < 0.001). Mobility evidenced explanatory power for participant scores on occupational activity and including this path improved model fit.

**Figure 2:**
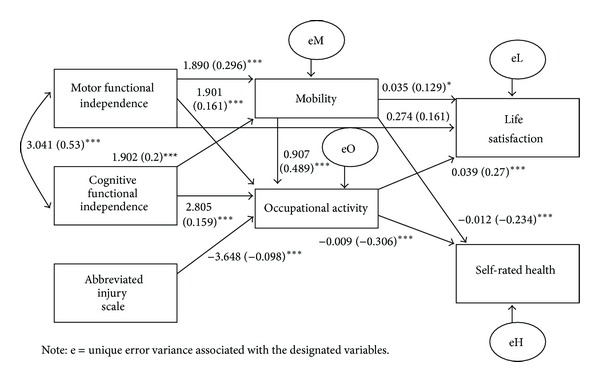
Corrected path model of motor functional independence, cognitive functional independence, abbreviated injury scale (head), mobility, occupational activity, life satisfaction, and self-rated health. Coefficients presented as unstandardized (standardized). *P < 0.05. ***P < 0.001.

The corrected model—displayed in Figure 2—was constructed with three exogenous variables (AIS, COG, and MOT), two mediating variables (MOB and OCC), and two endogenous outcome variables (LSI and HEALTH). This empirically derived model evidenced adequate model fit with the data *χ* ^2^ (8) = 6.074, *P* = 0.639 (RMSEA = 0.0, SRMR = 0.022). Accordingly, the model fit statistics provided evidence to support further interpretation of path coefficients in the corrected model.

#### 3.1.1. Direct Effects

The direct effects for the corrected model are shown in Table 2. Greater motor functional independence at 12-month post-discharge predicted greater mobility (*P* < 0.001) and occupational activity (*P* < 0.001) at 48-month post-discharge. Greater cognitive functional independence at 12-month post-discharge predicted greater mobility (*P* < 0.001) and occupational activity (*P* < 0.001) at 48-month post-discharge. Lower AIS head ratings (i.e., less severe head injuries) at time of injury were also predictive of greater occupational activity (*P* < 0.001) at 48-month post-discharge. 

 Greater mobility at 48-month post-discharge predicted greater life satisfaction (*P* < 0.05) and better self-rated health status (*P* < 0.001) at 60-month post-discharge. Additionally, mobility at 48-month post-discharge was predictively associated with greater occupational activity (*P* < 0.001) at 48-month post-discharge. Subsequently, greater occupational activity at 48-month post-discharge predicted greater life satisfaction (*P* < 0.001) and better self-rated health status (*P* < 0.001) at 60-month post-discharge. The model accounted for 19% of the variance in mobility (*R* ^2^ = 0.193), 44% of the variance in occupation activities (*R* ^2^ = 0.445), 21% of the variance in life satisfaction (*R* ^2^ = 0.21), and 23% of the variance in self-rated health (*R* ^2^ = 0.236). 

#### 3.1.2. Indirect Effects

All possible indirect effects on life satisfaction and self-rated health status at 60-month post-discharge were tested with the bootstrap procedure using the Mplus statistical software (see Table 3). The bootstrap procedure constructed 95% confidence intervals of indirect effects to test for statistical significance.

*Occupational Activity.* Occupational activity at 48-month post-discharge mediated the relationship of motor functional independence to life satisfaction (*P* < 0.05) and to self-rated health status (*P* < 0.001). Occupational activity at 48-month post-discharge also mediated the relationship of cognitive functional independence to self-rated health status (*P* < 0.01) and to life satisfaction (*P* < 0.05). Similarly, occupational activity mediated the relationship of AIS head ratings to both life satisfaction (*P* < 0.01) and to self-rated health status (*P* < 0.001). Thus, greater motor and cognitive functional independence at 12-month post-discharge and less injury were indirectly associated with increased life satisfaction and self-rated health status at 60-month post-discharge through their association with greater occupational activity at 48-month post-discharge.

*Mobility*. Mobility at 48-month post-discharge mediated the predictive relationship between cognitive functional independence at 12-month post-discharge and life satisfaction at 60-month post-discharge (*P* < 0.001). Additionally, the indirect effect between cognitive functional independence at 12-month post-discharge and self-rated health status at 60-month post-discharge was statistically significant (*P* < 0.001). Consequently, greater cognitive functional independence at 12-month post-discharge was predictive of greater life satisfaction and better self-rated health status at 60-month post-discharge though cognitive functional independence's association with mobility at 48-month post-discharge. 

## 4. Discussion

 Similar to the Erosa et al. [16] study, these results provide evidence that participation mediates the prospective relationship of functional impairment and severity to elements of quality of life five years after discharge for TBI. Although other work documents the importance of participation to quality of life following TBI [40], to our knowledge the present study is the first to demonstrate how participation can mediate the influence of impairment and injury severity on life satisfaction and self-rated health. 

 A recent meta-analysis of injury severity, functional impairment, and outcomes after TBI found that functional impairment (as measured by the FIM) had sizeable effect sizes in relation to “global disability,” but very low effect sizes were found between FIM scores and various measures of quality of life [1]. These authors concluded that the severity of functional impairments may be clinically useful in anticipating difficulties that impact long-term outcomes. Results from the present study suggest that these anticipated difficulties may be reflected, in part, in the prospective influence of functional impairment (and injury severity) on participation and in the subsequent mediating effects on quality of life. 

According to Hoyt et al. [41], when mediation occurs it “…speaks to the explanation for the mechanism that drives this relationship” (p. 323). Thus, the pattern of mediation in the present study suggests that greater impairment complicates an individual's mobility in the home and in the community and thwarts their ability to engage in meaningful, productive work-related and leisure activity. Injury severity also contributes to restrictions in work-related and leisure activity. In contrast, those with more functional abilities and less severe injuries are more likely to be mobile in their surroundings and engage in desired leisure and work-related activities. Participation in these domains, then, is predictive of greater life satisfaction and more optimal appraisals of health over time. The final model found no significant direct effects of functional impairment or injury severity to life satisfaction or to self-rated health. The association of functional impairment and injury severity with subsequent adjustment may be best understood in the context of their relationships with participation. Attention to these “indirect” effects might be more important than their assumed “direct” effects on measures of life satisfaction and self-rated health. 

 In the present study, mobility and occupational activity represented two dimensions of participation that exhibited a direct influence on adjustment. Interestingly, the final model indicated that mobility had a positive influence on the occupation variable. There is evidence that access to transportation is predictive of occupational activity among persons with moderate and severe TBI [42]. The beneficial association of mobility to occupational activity merits empirical scrutiny in future research. 

 However, it is important to recognize that the Occupation Scale on the CHART assesses activities associated with meaningful career-related roles including volunteerism, recreational pursuits, and time spent in school in addition to time in paid work. The scale also requires respondents to report the amount of time spent in household chores and home maintenance. Consequently, the scale may best reflect a purposeful “productivity” [42] that has clear benefits on an individual's sense of well-being and overall health. Our findings concerning the negative impact of cognitive and motor impairment on occupational activity are consistent with prior research [43]. Nevertheless, the scale confounds work-related activity with leisure and recreational pursuits. There is evidence that work-related activity is uniquely associated with greater quality of life among persons with TBI [44]. There is also a concern that rehabilitation professionals underestimate the value persons with TBI place on leisure activities [45]. Disentangling work-related activities from recreational and leisure pursuits in future research may provide valuable information about the benefits of each on quality of life after TBI. 

The pattern of relationships displayed in Figure 2 is consistent with our theoretical understanding of intentional activity and its potential impact on well-being following acquired disability [6, 11, 12]. With greater capacity to engage in intentional, purposeful activity, there is a greater likelihood that participation in meaningful social roles will ensue. Participation then increases the probability for rewarding interactions with others and for experiencing positive emotions in these interactions. These elements—supportive interactions with others and positive affect—are key ingredients in subjective well-being, life satisfaction, and happiness among people in general [13]. Nevertheless, the exclusion of Social Integration from the final model tempers this interpretation, and it may imply that the benefits from being mobile and engaging in purposeful activities may stem from the sense of competency, personal fulfillment, and attainment of personal goals that perpetuate positive affect and well-being. It is apparent from the correlations in Table 1 that Social Integration was significantly associated with life satisfaction and perceived health in expected directions. The final model suggests that Social Integration was not a significant influence on these outcomes, however, once the predictive values of the Mobility and Occupation variables were considered. 

The conceptual flow of the study variables in the final model provides insight into the ways in which personal adjustment may be enhanced in the years following TBI onset. Individuals who have greater freedom to move about in their communities and who can resume purposeful, productive activities are more likely than others to experience a higher quality of life. Community-based programs and services that promote personal mobility in the environment and that assist in resuming productive activities may facilitate personal adjustment over time. Individuals with severe injuries and more functional impairments encounter more difficulties in participation, and community-based programs that address these impediments and promote participation are indicated. 

The present study is limited in several respects. We relied on the AIS head ratings as the sole indicator of injury severity. Other, more commonly used variables (such as loss of consciousness, coma scores) were not available. Similarly, we relied on a single-item measure of health status. We know from prior research that individuals with mild, less severe injuries and those with substance abuse problems are often lost to follow-up [46]. Furthermore, our study was informed by current conceptualizations of adjustment that stem from the positive psychology literature [6, 7, 9, 11, 12]. Consequently, we were not interested in participant depression, anxiety, or other psychiatric disorders. These problems are adversely associated with quality of life following TBI [47]. We do not know the degree to which any of these factors might have influenced our results. 

We were interested in participation as a theoretically important mediating factor in the prediction of quality of life. Other important variables could also serve as important predictors (and potential mediators) in our model (e.g., pain, fatigue, executive functioning, and processing speed). We did not control for possible differences that might occur as a function of proxy reporting by family members. The current literature regarding this matter is mixed and without clear directives for the measures used in the present study [48–50]. We do not understand why the social integration variable failed to contribute to the model as originally hypothesized. Rather than minimize the role of this variable in adjustment, we suggest future research examining additional indicators of social integration to understand the features of this variable that contribute to quality of life following TBI.

## Figures and Tables

**Table 1:** Bivariate correlations, means, and standard deviations of variables in path model.

Variable	1	2	3	4	5	6	7	8	M	SD
(1) MOT	—	0.530**	0.022	0.402**	0.439**	0.183**	0.340**	−0.258**	6.009	2.927
(2) COG		—	−0.004	0.357**	0.419**	0.165**	0.300**	−0.263**	2.941	1.966
(3) AIS			—	−0.021	−0.104	−0.007	0.041	−0.018	2.910	0.933
(4) MOB				—	0.612**	0.315**	0.359**	−0.422**	87.410	18.676
(5) OCC					—	0.193**	0.420**	−0.449**	69.700	34.703
(6) SI						—	0.115*	−0.170**	80.130	17.571
(7) LSI							—	−0.459**	11.790	5.006
(8) Health								—	2.130	0.984

*P < 0.05, **P < 0.01.

**Table 2:** Direct effects of corrected path model.

Effect	Unstandardized estimate	Standardized estimate	SE	Critical ratio
MOT → MOB***	1.890	0.296	0.415	4.554
COG → MOB***	1.902	0.200	0.477	3.983
MOT → OCC***	1.901	0.161	0.347	5.483
COG → OCC***	2.805	0.159	0.588	4.768
AIS → OCC***	−3.648	−0.098	0.553	−6.598
MOB → OCC***	0.907	0.489	0.111	8.209
MOT → LSI	0.274	0.161	0.148	1.852
MOB → LSI*	0.035	0.129	0.015	2.281
OCC → LSI***	0.039	0.270	0.010	3.904
MOB → Health***	−0.012	−0.234	0.003	−3.583
OCC → Health***	−0.009	−0.306	0.002	−5.211

*P < 0.05. ***P < 0.001.

MOT: motor functional independence at 12-month post-discharge; COG: cognitive functional independence at 12-month post-discharge; AIS: Abbreviated Injury Scale head severity ratings obtained in chart review; MOB: mobility at 48-month post-discharge; OCC: occupational activities at 48-month post-discharge; LSI: life satisfaction inventory at 60-month post-discharge; Health: self-rated health status at 60-month post-discharge.

**Table 3:** Indirect effects of corrected path model.

Effect	Unstandardized estimate	Standardized estimate	SE	Crit ratio	95% CI
MOT → MOB → LSI	0.065	0.038	0.080	2.584	−0.013, 0.144
MOT → OCC → LSI*	0.074	0.043	0.031	2.370	0.013, 0.135
COG → MOB → LSI***	0.066	0.026	0.017	3.865	0.032, 0.099
COG → OCC → LSI*	0.109	0.043	0.048	2.291	0.016, 0.203
AIS → OCC → LSI**	−0.142	−0.027	0.050	−2.846	−0.240, −0.044
MOT → MOB → Health	−0.023	−0.069	0.012	−1.894	−0.047, 0.001
MOT → OCC → Health***	−0.016	−0.049	0.003	−6.329	−0.022, −0.011
COG → MOB → Health***	−0.023	−0.047	0.004	−6.408	−0.031, −0.016
COG → OCC → Health**	−0.024	−0.049	0.008	−3.067	−0.040, −0.009
AIS → OCC → Health***	0.032	0.030	0.003	11.749	0.026, 0.037

*P < 0.05, **P < 0.01, ***P < 0.001.

MOT: motor functional independence at 12-month post-discharge; COG: cognitive functional independence at 12-month post-discharge; AIS: Abbreviated Injury Scale head severity ratings obtained by chart review; MOB: mobility at 48-month post-discharge; OCC: occupational activities at 48-month post-discharge; LSI: life satisfaction inventory at 60-month post-discharge; Health: self-rated health status at 60-month post-discharge; Crit Ratio: critical ratio.
